# Carfilzomib with immunomodulatory drugs for the treatment of newly diagnosed multiple myeloma

**DOI:** 10.1038/s41375-019-0517-6

**Published:** 2019-07-24

**Authors:** Ola Landgren, Pieter Sonneveld, Andrzej Jakubowiak, Mohamad Mohty, Karim S. Iskander, Khalid Mezzi, David S. Siegel

**Affiliations:** 1 Memorial Sloan Kettering Cancer Center, New York, NY USA; 2 Erasmus MC Cancer Institute, Rotterdam, Netherlands; 3 University of Chicago Medicine, Chicago, IL USA; 4 Saint-Antoine Hospital, Sorbonne University, INSERM UMRs 938, Paris, France; 5 Amgen Inc., Thousand Oaks, CA USA; 6 John Theurer Cancer Center, Hackensack University Medical Center, Hackensack, NJ USA

**Keywords:** Cancer, Medical research

## Abstract

Carfilzomib, a selective proteasome inhibitor (PI), is approved for the treatment of patients with relapsed or refractory multiple myeloma (MM). Combination regimens incorporating a PI and immunomodulatory drug (IMiD) have been associated with deep responses and extended survival in patients with newly diagnosed MM (NDMM). Carfilzomib-based combinations with immunomodulators are being extensively studied in the frontline setting. The objective of this review was to describe efficacy and safety data for carfilzomib-based, PI/immunomodulatory combinations in NDMM. Information sources were articles indexed in PubMed and abstracts from key hematology/oncology congresses published between January 2012 and December 2018. PubMed and congresses were searched for prospective clinical studies assessing the combination of carfilzomib with an IMiD for NDMM treatment. Retrospective and preclinical reports, case reports/series, reviews, and clinical studies not evaluating carfilzomib–immunomodulator combinations in NDMM were excluded based on review of titles and abstracts. A total of nine articles and 72 abstracts were deemed relevant and included in the review. A total of six distinct carfilzomib-based, PI/immunomodulator combination regimens have been evaluated in 12 clinical trials. Overall, treatment with these regimens has resulted in deep responses, including high rates of negativity for minimal residual disease. These deep responses have translated to long progression-free survival and overall survival rates. Efficacy results for these regimens have generally been consistent across subgroups defined by age, transplant eligibility, and cytogenetic risk. The safety profile of carfilzomib in NDMM is consistent with that observed in the relapsed-refractory MM setting. Clinical studies have found that carfilzomib-based combinations with immunomodulators are highly active with a favorable safety profile in NDMM. The carfilzomib, lenalidomide, and dexamethasone (KRd) drug backbone is a promising foundation for treatment strategies aimed at achieving long-term, deep responses (functional cures) in the frontline setting. Several ongoing studies are evaluating KRd, with or without anti-CD38 monoclonal antibodies.

## Introduction

There were 138,509 new multiple myeloma (MM) cases worldwide in 2016; in the United States, 30,770 projected cases occurred in 2018 [1, 2]. The global incidence has increased sharply in recent decades, in part due to aging populations [1].

For newly diagnosed MM (NDMM) patients, the introduction of proteasome inhibitors (PIs) and immunomodulatory drugs (IMiDs) has improved treatment responses versus older therapies [3]. Several modern combination regimens incorporating a PI–IMiD backbone are considered recommended frontline regimens by the National Comprehensive Care Network and the European Society for Medical Oncology. These clinical regimens build off preclinical evidence showing that PIs and IMiDs have synergistic activity mediated by enhanced proteasome targeting, caspase activation, NF-κB inhibitory activity, and downregulation of CRBN/IRF4/MYC signaling and MCL1 [4–6]. These modern combination regimens have been associated with deep responses and improved progression-free survival (PFS) and overall survival (OS) [7].

**Fig. 1:**
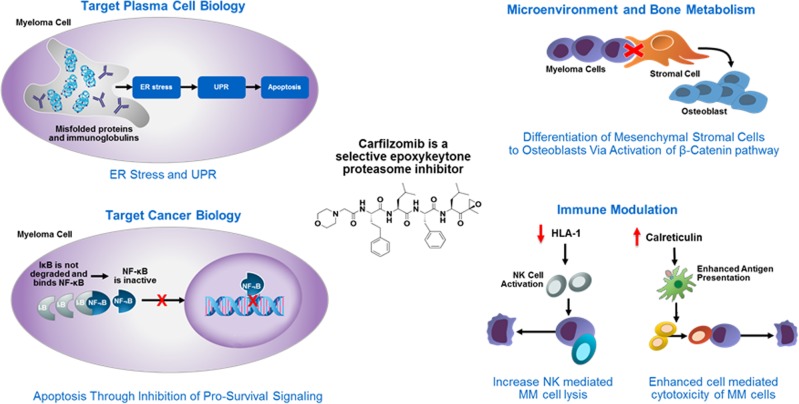
Mechanisms of action for carfilzomib [12–17]. ER endoplasmic reticulum, HLA human leukocyte antigen, MM multiple myeloma, NF-κB nuclear factor kappa-light-chain-enhancer of activated B cells, NK natural killer, UPR unfolded protein response

Carfilzomib is a selective, second-generation PI that irreversibly binds the proteasome [8, 9]. This agent exerts its antimyeloma activity through several actions including unfolded protein stress response induction [10], NF-κB prosurvival activity downregulation [11], modification of bone turnover and the bone marrow microenvironment leading to increased bone strength and compromised microenvironmental support for myeloma cells [12–14], and induction of immunogenic myeloma cell death through increased natural kill cell-mediated MM-cell lysis and enhanced antigen presentation (Fig. 1) [12–17]. Carfilzomib has a structure (tetrapeptide epoxyketone) differentiated from the first-generation PI bortezomib (dipeptide boronate) [8]. Preclinical studies demonstrated that carfilzomib overcomes bortezomib resistance [9] and that it does not reduce neurite length or inhibit nonproteasomal targets like HtrA2/Omi, effects which were observed with bortezomib [8]. Carfilzomib elicits deeper and more sustained proteasome inhibition versus bortezomib [18].

In MM clinical trials, carfilzomib-based regimens demonstrated robust efficacy and carfilzomib showed single-agent activity; it is approved for treatment of relapsed or refractory MM (RRMM). The approvals of carfilzomib-based combination therapy were based on three randomized phase 3 relapsed and/or refractory MM studies: ASPIRE, ENDEAVOR, and A.R.R.O.W. In ASPIRE and ENDEAVOR, treatment with carfilzomib-based regimens (ASPIRE: carfilzomib–lenalidomide–dexamethasone [KRd]; ENDEAVOR: carfilzomib–dexamethasone [Kd]) resulted in superior PFS, OS, depth of response, and health-related quality of life (QoL) versus recent standards of care in patients with RRMM [19–24]. In A.R.R.O.W., treatment with a more convenient once-weekly Kd regimen (carfilzomib 70 mg/m^2^) improved PFS and health-related QoL compared with twice-weekly Kd (carfilzomib 27 mg/m^2^) in patients with relapsed and refractory MM [25, 26].

Given the established efficacy of carfilzomib in RRMM and the frontline potency of regimens incorporating a PI–IMiD backbone, recent NDMM clinical trials have evaluated combination regimens using carfilzomib-based, PI–IMiD combinations. This systematic review summarizes the efficacy and safety of these regimens for NDMM.

## Methods

**Fig. 2:**
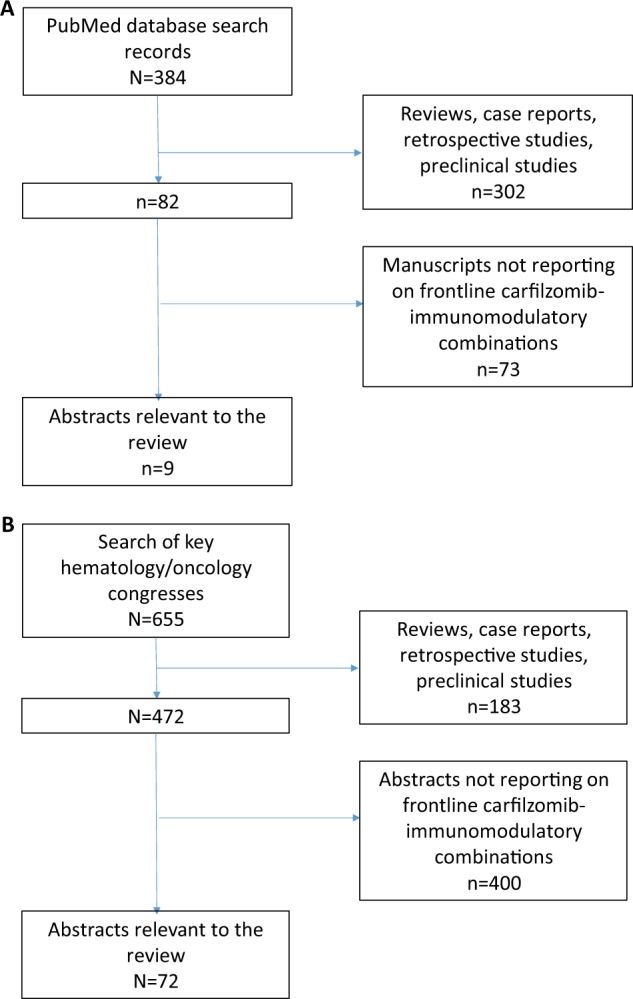
Identification of relevant manuscripts (**a**) and congress abstracts (**b**). Duplicates and preclinical publications were filtered

Studies were collected from a search of PubMed and abstracts from key hematology/oncology congresses published between January 2012 and December 2018. The following congresses were systemically searched between January 2012 and December 2018: American Society of Hematology Annual Meeting, American Society of Clinical Oncology Annual Meeting, European Hematology Association Annual Congress, Blood and Marrow Transplantation Tandem Meeting, European Society for Medical Oncology Annual Congress, International Myeloma Workshop, and the Lymphoma & Myeloma Congress. PubMed and congresses were searched for prospective clinical studies that assessed the combination of carfilzomib with an IMiD in NDMM patients. Specific search terms included “carfilzomib,” “Kyprolis,” “MM,” “thalidomide,” “lenalidomide,” “pomalidomide,” “newly diagnosed,” “transplant-eligible,” and “transplant-ineligible.” Three hundred and eighty-four articles were identified and were screened for relevancy to this review based on titles and abstracts. Retrospective and preclinical reports, case reports/series, reviews, and clinical studies not evaluating frontline carfilzomib-immunomodulator combinations were excluded. Nine articles were deemed relevant for inclusion (Fig. 2a). Six hundred and fifty-five abstracts were identified, 72 of which were deemed relevant (Fig. 2b).

## Results

### Activity of carfilzomib–IMiD combinations in NDMM

To date, six distinct carfilzomib–IMiD combinations have been assessed in 12 NDMM clinical trials [27–51]. Key study information and efficacy data (including minimal residual disease [MRD] and MRD limit of detection) are summarized in Tables 1 and 2 and described below. All studies assessed twice-weekly carfilzomib unless stated otherwise.

**Table 1:** Carfilzomib-immunomodulatory drug combination studies in patients with newly diagnosed multiple myeloma

Study	Study design	Primary endpoint	*N*	Patient population	Treatment	Quality of evidence rating^a^
**KRd**						
MMRC (KRd with or without ASCT) [27]	Open-label, phase 1/2; TITE-CRM method for dose-escalation	Phase 1: safety and MTD of carfilzomib; Phase 2: Rate of ≥nCR after 4 cycles	53	Transplant-eligible and -ineligible NDMM	KRd induction in 28-day cycles for up to 8 cycles or until PD or unacceptable toxicity; KRd maintenance for cycles 9–24; Single-agent LEN recommended after cycle 24; Carfilzomib (20, 27, or 36 mg/m^2^; 5- to 10-min IV infusion for 20 or 27 mg/m^2^ doses; 30 min for 36 mg/m^2^) on days 1, 2, 8, 9, 15, and 16 (1, 2, 15, and 16 during maintenance); LEN 25 mg on days 1–21; DEX 40 mg (20 mg after cycle 4) on days 1, 8, 15, and 22; ASCT was optional	2
KRd + LEN maintenance [30]	Clinical and correlative pilot study	NDMM: grade ≥3 neuropathy; SMM: ≥VGPR rate	57	NDMM (*n* = 45) or high-risk SMM (*n* = 12)	KRd for up to 8 28-day cycles. For patients with SD or better, LEN maintenance for up to 2 years; Carfilzomib (36 mg/m^2^; 30-min IV infusion) on days 1, 2, 8, 9, 15, and 16; LEN 25 mg on days 1–21 (not given on day 1 of cycle 1); DEX 20 mg (cycles 1–4) or 10 mg (cycles 5–8) on days 1, 2, 8, 9, 15, 16, 22, and 23 (not administered on day 1 of cycle 1); ASCT-eligible patients underwent SCC after cycle 4	2
MMRC (KRd + ASCT) [34]	Phase 2	Rate of sCR at end of cycle 8	76	Transplant- eligible NDMM (no age limitations)	Four cycles of KRd induction followed by ASCT, 4 cycles of KRd consolidation, and 1 year of KRd maintenance; Carfilzomib (36 mg/m^2^; 30-min IV infusion) on days 1, 2, 8, 9, 15, and 16 (1, 2, 15, and 16 for maintenance); LEN 25 mg (15 mg in cycle 5) on days 1–21; DEX 40 mg/week (20 mg/week during consolidation)	2
IFM study; KRd + ASCT + LEN maintenance [35]	Open-label, phase 2	Rate of sCR at end of consolidation	46	Transplant-eligible NDMM, age <65 years	Four cycles of KRd induction followed by ASCT, 4 cycles of KRd consolidation, and 1 year of LEN maintenance; Carfilzomib (36 mg/m^2^; 30-min IV infusion) on days 1, 2, 8, 9, 15, and 16; LEN 25 mg on days 1–21 (10 mg during maintenance); DEX 20 mg on days 1, 2, 8, 9, 15, 16, 22, and 23 (days 1, 8, 15, and 22 for consolidation)	2
MRD response-driven KRd [36]	Phase 1/2, 3 + 3 dose-escalation schema	Rate of MRD-negativity at the MTD	29	NDMM	KRd in 28-day cycles for a maximum of 12 cycles; MRD-negativity after any cycle: 2 additional KRd cycles before discontinuation; MRD-positive after any cycle: continued KRd until treatment completion, PD, or unacceptable toxicity; Carfilzomib (45 or 56 mg/m^2^) on days 1, 2, 8, 9, 15, and 16; LEN 25 mg on days 1–21; DEX 40 mg/week (20 mg/week after cycle 4)	2
FORTE; KRd versus KCd [38]	Randomized, phase 2	≥VGPR rate after induction	474	Transplant-eligible NDMM, age <65 years	Arm A: 4 cycles KCd induction followed by ASCT and 4 cycles of KCd consolidation; Arm B: 4 cycles of KRd induction followed by ASCT and 4 cycles of KRd consolidation; Arm C: 12 cycles of KRd; All arms randomized to LEN or carfilzomib-LEN maintenance; Carfilzomib 36 mg/m^2^ on days 1, 2, 8, 9, 15, and 16 of a 28-day cycle; LEN 25 mg on days 1–21; DEX 20 mg on days 1, 2, 8, 9, 15, and 16); Cyclophosphamide 300 mg/m^2^ on days 1, 8, and 15	1
**KTd**						
CARTHADEX (KTd) [41]	Open-label, phase 2	≥VGPR rate after induction	91	Transplant-eligible NDMM, age 18–65 years	4 cycles KTd induction followed by ASCT and 4 cycles KTd consolidation; Carfilzomib 27, 36, 45, or 56 mg/m^2^ on days 1, 2, 8, 9, 15, and 16 of a 28-day cycle; THAL 200 mg (induction) or 50 mg (consolidation) on days 1–28; DEX 20 mg on days 1, 2, 8, 9, 15, and 16	2
**CYKLONE**						
CYKLONE (Cyclophosphamide + KTd) [44]	Open-label, phase 1b/2, 3 + 3 dose-escalation schema	Phase 1: MTD of carfilzomib; Phase 2: ≥VGPR rate after 4 cycles	64	Transplant-eligible NDMM	CYKLONE for ≥4 cycles followed by ASCT; patients with ≥SD up to 8 additional cycles; Carfilzomib 20, 27, 36, or 45 mg/m^2^ (30-min IV infusion) on days 1, 2, 8, 9, 15, and 16 of a 28-day cycle; THAL 100 mg on days 1–28; DEX 40 mg on days 1, 8, 15, and 22; Cyclophosphamide 300 mg/m^2^ on days 1, 8, and 15	2
**Car-BiRD**						
Car-BiRD [47]	Phase 2	Not reported	72	Transplant-eligible and -ineligible NDMM	Kd until CR or stable M-protein for 2 consecutive cycles (maximal response), then SCC for ASCT-eligible patients, then BiRD consolidation until maximal response followed by LEN maintenance; Carfilzomib 45 or 56 mg/m^2^ (30-min IV) on days 1, 2, 8, 9, 15, and 16 of a 28-day cycle; DEX 20 mg on carfilzomib days (for Kd) or 40 mg on days 1, 8, 15, and 22 (for BiRD); LEN 25 mg on days 1–21 (for BiRD) or 10 mg for maintenance; Clarithromycin 500 mg twice daily	2
**KRd plus daratumumab**						
MMY1001 (KRd+DARA) [49]	Open-label, phase 1b	Safety and tolerability	22	Transplant-eligible and -ineligible NDMM	KRd + DARA for up to 13 cycles or discontinuation due to ASCT; Carfilzomib 70 mg/m^2^ on days 1, 8, and 15; LEN 25 mg on days 1–21; DEX 40 mg/week; DARA 16 mg/kg weekly (cycles 1 and 2), every 2 weeks (cycles 3–6), or every 4 weeks (cycles 7+)	2
NCT03290950 (KRd+DARA) [51]	Phase 2, single-arm, clinical and correlative, Simon’s optimal two-stage design	MRD-negativity	Two cohorts, *n* = 41 each	NDMM	KRd + DARA for eight 28-day cycles; Cohort 1: Carfilzomib 36 mg/m^2^ on days 1, 2, 8, 9, 15, and 16 Cohort 2: Carfilzomib 56 mg/m^2^ on days 1, 8, and 15; Cohorts 1 and 2: LEN 25 mg on days 1–21; DEX 40 mg weekly (20 mg after cycle 4); DARA 16 mg/kg on days 1, 8, 15, and 22 for cycles 1–2, days 1 and 15 for cycles 3–6, and day 1 for cycles 7–8	2
**KCRd**						
UK NCRI Myeloma XI Phase 3 trial (KCRd versus CRd or CTd) [48]	Phase 3, open-label, randomized	PFS and OS for each randomization	1056	Transplant-eligible or -ineligible NDMM, no age limits	Induction treatment with KCRd, CTd, or CRd induction was for a minimum of 4 cycles and continued until maximal response or intolerance; In the CTd and CRd arms, patients with SD/PD as maximum response received CVd as second induction, those with MR/PR were randomized to CVd or nothing as second induction, and those with VGPR + proceeded to ASCT Cycles were 21 days for CTd and CVd and 28 days for CRd and KCRd Carfilzomib 36 mg/m^2^ on days 1, 2, 8, 9, 15, and 16 THAL 100–200 mg on days 1–21; DEX 40 mg on days 1–4 and 12–15 (CTd, CRd, and CVd) or days 1–4, 8–9, and 15–16 (KCRd) Cyclophosphamide 500 mg on days 1, 8, and 15 (CTd arm only); LEN 25 mg on days 1–21	1

*ASCT* autologous stem cell transplantation, *Car-BiRD* carfilzomib induction with lenalidomide and clarithromycin consolidation and lenalidomide maintenance, *CYKLONE* cyclophosphamide, carfilzomib, thalidomide and dexamethasone, *CR* complete response, *CRd* cyclophosphamide, lenalidomide, and dexamethasone, *CTd* cyclophosphamide, thalidomide, and dexamethasone, *CVd* cyclophosphamide, bortezomib, and dexamethasone, DARA, daratumumab, *DEX* dexamethasone, *IFM* Intergroupe francophone du myélome, *IV* intravenous, *KCRd* carfilzomib, cyclophosphamide, lenalidomide, and dexamethasone, *KRd* carfilzomib, lenalidomide, and dexamethasone, *KTd* carfilzomib, thalidomide, and dexamethasone, *LEN* lenalidomide, *MMRC* Multiple Myeloma Research Consortium, *MR* minimal response, *MRD* minimal residual disease, *MTD* maximum tolerated dose, *NDMM* newly diagnosed multiple myeloma, *NIH* National Institutes of Health, *OS* overall survival, *PD* progressive disease, *PFS* progression-free survival, *PR* partial response, *sCR* stringent complete response, *SD* stable disease, *THAL* thalidomide, *TITE-CRM* time-to-event continual reassessment method, *UK NCRI* United Kingdom National Cancer Research Institute, *VGPR* very good partial response

^a^The rating scheme is as follows: 1: properly powered and conducted randomized clinical trial; 2: Well-designed controlled trial without randomization

**Table 2:** Activity of carfilzomib-immunomodulatory drug combination therapies in patients with newly diagnosed multiple myeloma

Study	*N*	Patient population	Best response (%)	Response (≥CR) by transplant status (%)		Response (≥CR) by age		Response (≥CR) by cytogenetics		MRD negativity,%	Median PFS, months	PFS rate, %	Median OS, months	OS rate,%
				Proceed with ASCT	Did not proceed with ASCT	≥65 years	<65 years	High risk	Standard risk					
**KRd**														
MMRC (KRd with or without ASCT) [27]	53	Transplant-eligible and -ineligible NDMM	≥VGPR: 81; ≥nCR: 62; sCR: 42	29 (before ASCT)	67 (≥nCR)	Not reported	Not reported	65 (≥nCR)	59 (≥nCR)	91 (10-color MFC; assessed in patients with CR/suspected CR)^a^	Not reported	97 (12 months) 92 (24 months)	Not reported	Not reported
Additional follow-up of MMRC study (KRd with or without ASCT) [29]	53	Transplant- eligible and -ineligible NDMM	sCR: 51 (end of cycle 18)	Not reported	Not reported	Not reported	Not reported	Not reported	Not reported	Not reported	Not reported	92 (24 months); 69 (48 months)	Not reported	Not reported
MMRC subgroup analysis (KRd in elderly) [28]	23	Transplant-eligible and -ineligible NDMM, age ≥65 years subset	≥VGPR: 91; ≥CR: 79; sCR: 65	Not reported	Not reported	79	Not reported	Not reported	Not reported	86 (10-color MFC; assessed in patients with sCR/nCR)^a^	Not reported	79.6 (36 months)	Not reported	100 (36 months)
KRd + LEN maintenance [30]	57	NDMM (45) or high-risk SMM (12)	≥VGPR: 89 (NDMM); 100 (SMM); ≥nCR: 62 (NDMM); 100 (SMM); ≥CR: 56 (NDMM); 100 (SMM)	Not reported	Not reported	Not reported	Not reported	Not reported	Not reported	NDMM: 100 (8-color MFC)^b^ 67 (NGS)^a^; SMM: 92 (8-color MFC)^b^ 75 (NGS)^a^ Assessed in patients with ≥nCR	Not reached	NDMM: 95 (12 month), 92 (18 month); SMM: 100	Not reported	Not reported
KRd + LEN maintenance: 5-year follow-up [32]	45	NDMM	≥PR: 98; ≥CR: 67	Not reported	Not reported	74	62	67	67	Percentage of patients (*n* = 45) with MRD-negative CR: 62 (MFC)^b^	67.3 (time to progression)	Not reported	Not reached	84 (6 years)
MMRC (KRd + ASCT) [34]	76	Transplant- eligible NDMM	≥VGPR: 91; ≥CR: 78; sCR: 75	Not reported	Not reported	Not reported	Not reported	Not reported	Not reported	Cycle 8: 67 (NGS)^c^ 95 (MFC)^d^; Cycle 18: 72 (NGS)^c^ 96 (MFC)^d^; After 1 year of LEN maintenance: 82 (NGS)^c^ 90 (MFC)^d^ MRD-negative status required CR	Not reported	86 (36 months); 94 for patients achieving MRD-negativity at end of cycle 18	Not reported	93 (36 months); 100 for patients with sustained MRD-negativity
IFM study; KRd++ASCT+LEN maintenance [35]	46	Transplant-eligible NDMM, age <65 years	Completion of consolidation: ≥VGPR: 85; ≥CR: 61	N/A	N/A	Not reported	Not reported	Not reported	Not reported	70 (MFC; assessed in patients with ≥ VGPR)^d^	Not reached	91 (24 months)	Not reported	Not reported
MRD response-driven KRd [36]	29	NDMM	VGPR: 40; ≥CR: 60; Response rates calculated for 15 patients who completed therapy	Not reported	Not reported	Not reported	Not reported	Not reported	Not reported	60 (MFC)^c^	Not reported	Not reported	Not reported	Not reported
FORTE; KRd versus KCd [38]	474	Transplant-eligible NDMM, age <65 years	Premaintenance response rates: ≥VGPR: 89 (KRd + ASCT) versus 76 (KCd + ASCT) versus 87 (KRd 12 cycles, no ASCT); sCR: 44 (KRd + ASCT) versus 32 (KCd + ASCT) versus 43 (KRd 12 cycles, no ASCT);	N/A	N/A	Not reported	Not reported	Not reported	Not reported	Premaintenance rates (ITT analysis): 58 (KRd + ASCT) versus 42 (KCd + ASCT) versus 54 (KRd 12 cycles, no ASCT) by second-generation MFC^b^	Not reported	Not reported	Not reported	Not reported
**KTd**														
CARTHADEX (KTd) [41]	91	Transplant-eligible NDMM, age 18–65 years	Response after induction: ≥VGPR: 68; CR: 25; Response after consolidation: ≥VGPR: 89; CR: 63	N/A	N/A	N/A	N/A	After consolidation: 66	After consolidation: 58	Not reported	Not reached	72 (36 months)	Not reported	Not reported
**CYKLONE**														
CYKLONE (Cyclophos+KTd) [44]	64	Transplant- eligible NDMM	All cycles: ≥VGPR: 69; ≥CR: 8; sCR: 3	73.5	Not reported	Not reported	Not reported	Not reported	Not reported	Not reported	Not reported	85 (12 months) 76 (24 months)	Not reported	96 (12 and 24 months)
**Car-BiRD**														
Car-BiRD [47]	72	Transplant-eligible and -ineligible NDMM	≥VGPR: 84; ≥CR: 37	Not reported	Not reported	Not reported	Not reported	Not reported	Not reported	Not reported	Not reported	Not reported	Not reported	Not reported
**KRd plus daratumumab**														
MMY1001 (KRd + DARA) [49]	22	Transplant-eligible and -ineligible NDMM	VGPR: 33; CR: 14; sCR: 43	Not reported	VGPR: 27; CR: 20; sCR: 40 (*n* = 15)	Not reported	Not reported	Not reported	Not reported	Not reported	Not reported	95 (12 months)	Not reported	All patients alive at time of follow up
NCT03290950 (KRd+DARA) [51]	20 (completed ≥1 cycle)	NDMM	All 3 patients who completed full assessments with MRD assays had a CR	Not reported	Not reported	Not reported	Not reporter	Not reported	Not reported	3 patients assessed: Patient 1: MRD negative in bone marrow and peripheral blood; Patient 2: MRD positive in bone marrow and MRD positive in peripheral blood; Patient 3: MRD negative in bone marrow and MRD positive in peripheral blood	Not reported	Not reported	Not reported	Not reported
**KCRd**														
UK NCRI Myeloma XI Phase 3 trial (KCRd versus CRd or CTd) [48]	1056	Transplant eligible NDMM	End of first induction ≥VGPR: 82.3 (KCRd) versus 52.8 (CTd) versus 64.9 (CRd); CR: 17.7 (KCRd) versus 6.8 (CTd) versus 7.1 (CRd); After ASCT: VGPR: 91.9 (KCRd) versus 76.1 (CTd) versus 82.1 (CRd); CR: 31.0 (KCRd) versus 25.2 (CTd) versus 22.9 (CRd)	Not reported	N/A	Not reported	Not reported	Not reported	Not reported	Post induction: 67 (KCRd) versus 19 (CTd) versus 18 (CRd); Post ASCT: 77 (KCRd) versus 53 (CTd) versus 56 (CRd) (8-color MFC^b^)	Not reported	64.5 (KCRd) versus 50.3 (CTd/CRd) (3 years)	Not reported	Not reported

*ASCT* autologous stem cell transplantation, *Car-BiRD* carfilzomib induction with lenalidomide and clarithromycin consolidation and lenalidomide maintenance, *CYKLONE* cyclophosphamide, carfilzomib, thalidomide and dexamethasone, *CR* complete response, *DEX* dexamethasone, *IFM* Intergroupe francophone du myélome, *IV* intravenous, *KCRd* carfilzomib, cyclophosphamide, lenalidomide, and dexamethasone, *KRd* carfilzomib, lenalidomide, and dexamethasone, *KTd* carfilzomib, thalidomide, and dexamethasone, *LEN* lenalidomide, *MFC* multiparametric flow cytometry; *MMRC* Multiple Myeloma Research Consortium, *MRD* minimal residual disease, *NDMM* newly diagnosed multiple myeloma, *NGS* next generation sequencing, *NIH* National Institutes of Health, *OS* overall survival, *PD* progressive disease, *PFS* progression-free survival, *sCR* stringent complete response, *UK NCRI* United Kingdom National Cancer Research Institute, *VGPR* very good partial response

^a^Sensitivity not reported

^b^Sensitivity of 1 × 10^−5^

^c^Sensitivity of 10^−5^−10^−6^

^d^Sensitivity of 10^−4^−10^−5^

#### Carfilzomib–lenalidomide–dexamethasone

In NDMM, KRd has been examined in six distinct clinical studies. In 2012, the MM Research Consortium (MMRC) reported findings from a phase 1/2 study of KRd (*n* = 53; transplant-eligible or -ineligible) [27]. Patients received KRd induction until disease progression or unacceptable toxicity for up to eight cycles (carfilzomib 20, 27, or 36 mg/m^2^ in phase 1; 36 mg/m^2^ in phase 2). Transplant-eligible patients who achieved ≥partial response (PR) after cycle 4 had stem cell collection (SCC) and the option to proceed with autologous stem cell transplantation (ASCT), but per protocol, were to resume KRd after SCC. After eight cycles, patients received KRd maintenance for a maximum of 24 total KRd cycles. After four cycles, 38% of patients achieved ≥near complete response (nCR; defined as no detectable M protein on electrophoresis independent of immunofixation status, stable bone disease, and normal serum calcium concentration; phase 2 primary endpoint). At a median follow-up of 13 months (median treatment duration, 12 cycles), overall response rate (ORR) was 98% including 42% with a stringent complete response (sCR). Approximately two-thirds of patients who completed ≥8 cycles achieved sCR. Among patients with a complete response (CR/suspected CR), the MRD-negativity rate was 91% (multiparametric flow cytometry [MFC]). Notably, deep responses were obtained with KRd alone as the seven patients who proceeded to ASCT were censored for best response at the time of proceeding to transplant because they did not resume KRd. PFS rates were 97% and 92% at 12 and 24 months, respectively. There were no major efficacy differences between standard- and high-risk cytogenetics subgroups, although numbers were limited (standard risk, *n* = 34; high risk, *n* = 17). In a subgroup analysis of the MMRC study evaluating elderly patients (age ≥65 years; *n* = 23), the ≥very good PR (VGPR) rate was 91%; the sCR rate was 65% [28]. Updated MMRC study results (median follow-up, 4 years) showed that extended KRd treatment increased sCR rates from 42 to 51% [29]. The 2- and 4-year PFS rates were 92% and 69%, respectively [29].

A phase 2 study National Institutes of Health (NIH) Clinical Center study evaluated KRd in transplant-eligible or -ineligible patients with NDMM (*n* = 45) or smoldering MM (SMM) at high risk of progression (*n* = 12) [30]. Patients received eight KRd cycles then lenalidomide maintenance. Carfilzomib (36 mg/m^2^) was administered for the first 3 weeks of a 4-week cycle. Deep responses were observed, with 62% (NDMM) and 100% (SMM) of patients achieving ≥nCR [30]. The 1-year PFS rate in NDMM was 95% and all NDMM patients remained alive [30]. Thirty-four of 45 enrolled NDMM patients (76%) achieved MRD-negative status (MFC). MRD-negativite ≥nCR rates by MFC were 100% (NDMM) and 92% (SMM); MRD-negativity rates by next-generation sequencing (NGS) were 67% and 75%, respectively [30]. MRD negativity was observed In hematopoietic progenitor cell grafts from 29/30 patients [31]. In a 5-year follow-up of the study (NDMM cohort), the MRD-negative CR rate was 62%; median time to progression was 67.3 months and the 6-year OS rate was 84% [32]. Depth of response and PFS were consistent regardless of age (≥65 vs <65 years) or cytogenetic risk [30, 32]. The NIH Clinical Center study was recently reopened for enrollment with expanded sample size. Based on results from the MMRC and NIH Clinical Center studies, KRd is a treatment option for transplant-eligible and -ineligible patients according to the National Comprehensive Care Network guidelines.

Although extended KRd treatment without transplant in the MMRC study showed strong clinical benefit, some patients experienced progression [27]. Therefore, a second phase 2 MMRC study added ASCT to KRd to evaluate if further improvement of outcomes was possible [33, 34]. In this study (*n* = 76), patients received four 28-day cycles of KRd induction followed by ASCT. Following ASCT, patients received four cycles of KRd consolidation and then KRd maintenance for 10 cycles. Carfilzomib was administered at 20/36 mg/m^2^. Response rates were 91% ≥VGPR, 78% ≥CR, and 75% sCR (primary endpoint) [33, 34]. Postconsolidation MRD-negative ≥CR rates were 67% (NGS) and 95% (MFC) [34]. High rates of MRD negativity after consolidation were found to be sustained through to cycle 18 and after lenalidomide maintenance; this sustained MRD negativity (cycle 18) correlated with 3-year PFS and OS rates [34]. The 3-year PFS and OS rates for sustained MRD negativity patients by NGS were 94 and 100%, which were higher than those in the overall population (PFS, 86%; OS, 93%) [34]. Similar findings were observed in patients with high-risk disease (*n* = 27) [34].

The Intergroupe Francophone du Myelome also conducted a phase 2 study evaluating KRd with ASCT [35]. Patients received four KRd induction cycles, ASCT, four KRd consolidation cycles, and 1-year lenalidomide maintenance. Carfilzomib (20/36 mg/m^2^) was given for 3 weeks of each 4-week cycle. Postconsolidation ORR was 89%, including 85% ≥VGPR and 61% ≥CR. MRD-negativity rate was 70% by flow cytometry. Median PFS was not reached; the 2-year PFS rate was 91%.

The high rates of MRD negativity observed with KRd and integration of MRD assessments into standardized response criteria [3] provided rationale for an ongoing phase 1/2 study that is the first to evaluate MRD status as a guide for KRd treatment duration in NDMM patients [36]. During phase 1, patients receive carfilzomib (45 or 56 mg/m^2^) with lenalidomide–dexamethasone to determine the maximum tolerated dose (MTD) of carfilzomib. Patients who achieve MRD negativity after any cycle receive two additional KRd cycles before treatment discontinuation, whereas MRD-positive patients continue therapy for ≤12 cycles, or until disease progression or unacceptable toxicity. The phase 2 primary endpoint is MRD negativity at the MTD. Phase 1 results established the carfilzomib MTD at 56 mg/m^2^. The phase 2 portion of the study is ongoing.

The demonstrated activity of KRd and the promising efficacy of carfilzomib–cyclophosphamide–dexamethasone (KCd) [37] in NDMM patients provided the rationale for the randomized FORTE trial, where NDMM patients were randomized 1:1:1 to four 28-day KCd cycles followed by ASCT and four KCd consolidation cycles; or four 28-day KRd cycles followed by ASCT and four KRd consolidation cycles; or twelve KRd cycles [38]. Patients who complete consolidation are randomized to lenalidomide versus carfilzomib–lenalidomide maintenance. Carfilzomib was administered at 20/36 mg/m^2^. A total of 474 patients were randomized (KRd + ASCT, *n* = 158; KCd+ASCT, *n* = 159; KRd 12 cycles, *n* = 157). Premaintenance, ≥VGPR and sCR rates were higher in the KRd arms than in the KCd arm (KRd + ASCT, 89 and 44%; KCd + ASCT, 76 and 32%; KRd 12 cycles, 87 and 43%). MRD-negativity rates were also higher with KRd versus KCd (KRd + ASCT, 58%; KCd + ASCT, 42%; KRd 12 cycles, 54%). These benefits for KRd versus KCd were consistent, regardless of the presence of high-risk disease. The sCR rates in the KRd arms also compare favorably with the postconsolidation sCR rate (28.9%) reported for the daratumumab–bortezomib–thalidomide–dexamethasone arm in the phase 3 CASSIOPEIA study of this combination in transplant-eligible NDMM [52].

Inspired by the NIH Clinical Center study, the ongoing phase 2 GEM-CESAR trial is assessing KRd induction and consolidation with ASCT for SMM at high risk of progression. Preliminary post-ASCT efficacy results (69% ≥CR; 58% MRD negative) demonstrate the promise of this approach [39]. An EMN-MSKCC randomized phase 2 study comparing KRd versus Rd in high-risk SMM has recently started.

Overall, the safety profile of KRd in NDMM patients has been consistent with the profile observed in the ASPIRE study. In the FORTE trial, rates of treatment discontinuation due to adverse events (AEs) and fatal AEs were 8 and 1%, whereas in ASPIRE these rates were 15% and 7%, respectively (see Tables 3 and 4 for additional details) [24, 38, 40].

**Table 3:** Safety of carfilzomib–immunomodulatory drug combination therapies in patients with newly diagnosed multiple myeloma

Study (reference)	Phase	Patient population	Safety population, *n*	Common any-grade AEs	Common grade ≥3 AEs	SAEs, %	Discontinuations due to AEs, (%)	Dose reductions due to AEs, (%)
**KRd**								
MMRC (KRd with or without ASCT) [27]	1/2	Transplant-eligible and -ineligible NDMM	53	Most common during induction: hyperglycemia (72%), thrombocytopenia (68%), anemia (60%), edema (47%), hypophosphatemia (45%), and fatigue (38%)	Hematologic (induction): anemia (21%), thrombocytopenia (17%), and neutropenia (17%); Nonhematologic (induction): hypophosphatemia (25%), hyperglycemia (23%), DVT/PE (9%), rash (8%), and elevated liver function test (8%).	Not reported	1.9 (*n* = 1 during induction)	Dose modifications during induction: 31
MMRC subset analysis [28]	1/2	Transplant-eligible and -ineligible NDMM, age ≥65 years	23	Not reported	Hematologic (induction): thrombocytopenia (39%), lymphopenia (35%), neutropenia (30%), and anemia (26%); Nonhematologic (induction, >10%): hyperglycemia (39%), hypophosphatemia (22%), and thromboembolic events (13%)	Not reported	4.3 (*n* = 1 during induction)	Dose modifications during induction: 74 (CFZ), 83 (LEN), 70 (DEX); Dose modifications during maintenance: 52 (CFZ), 57 (LEN), 61 (DEX)
MMRC (KRd+ASCT) [33]	2	Transplant-eligible NDMM	76	Hematologic KRd-related: thrombocytopenia (57%), lymphopenia (39%), anemia (39%), and neutropenia (28%); Nonhematologic KRd-related: fatigue (53%), peripheral neuropathy (39%), infection (34%), and diarrhea (3%)	Most common were lymphopenia (28%), neutropenia (18%), and infections (8%)	Not reported	Not reported	Not reported
KRd+LEN maintenance [30]	2	Transplant-eligible and -ineligible NDMM	45	The most common any-grade hematologic AE was lymphopenia (100%); the most common nonhematologic AEs were electrolyte or metabolism abnormalities (98%)	Not reported	Not reported	2.2 (*n* = 1)	Dose modifications: 44
IFM study; KRd+ASCT+LEN maintenance [35]	2	Transplant-eligible NDMM, age <65 years	46	Not reported	The most common grade 3/4 AEs after induction were infections (*n* = 7), neutropenia (*n* = 6), lymphopenia (*n* = 5), musculoskeletal disorders (*n* = 5), and cardiovascular disorders (*n* = 5);	65	Not reported	Not reported
MRD response-driven KRd [36]	1/2	NDMM	29	Not reported	Nonhematologic: rash (21%), electrolyte disturbances (17%), infections (14%), gastrointestinal (10%), cardiopulmonary (7%), venous thromboembolism (7%), mood (7%), cataract (7%), and hyperglycemia (3%); Hematologic: lymphopenia (41%), leukopenia (7%), neutropenia (3%), and thrombocytopenia (3%)	34	One patient came off study due to MI and another due to intolerable rash	Not reported
FORTE; KRd versus KCd [38]	3	Transplant-eligible NDMM, age <65 years	KRd+ASCT: 158; KRd 12 cycles, no ASCT: 157 KCd+ASCT: 159	Not reported	Rate of grade 3/4 or serious hematologic AEs related to KRd or KCd: 22% (KRd+ASCT) versus 17% (KCd+ASCT) versus 18% (KRd 12 cycles, no ASCT); Rate of grade 3/4 or serious nonhematologic AEs: 35% (KRd+ASCT) versus 26% (KCd+ASCT) versus 48% (KRd 12 cycles, no ASCT);	Not reported	8 (KRd+ASCT) versus 7 (KCd+ASCT) versus 8 (KRd 12 cycles, no ASCT)	32 (KRd+ASCT) versus 19 (KCd+ASCT) versus 36 (KRd 12 cycles, no ASCT) (Patients who reduced at least 1 drug)
**KTd**								
Carthadex [41]	2	Transplant-eligible NDMM, age 18–65 years	91	Blood and lymphatic system disorders: 16% (over all cycles)	Blood and lymphatic system disorders: 7%; Dermatologic: 10%; Respiratory: 15%; Gastrointestinal: 12% (over all cycles)	40	5	Carfilzomib dose reductions: 5
**CYKLONE**								
CYKLONE [44]	1b/2	Transplant-eligible NDMM	64	Hematologic: neutropenia (55%), thrombocytopenia (47%), anemia (44%), lymphopenia (42%) and leukopenia (39%); Nonhematologic: fatigue (80%), constipation (53%), hyperglycemia (39%) and lethargy (25%)	Hematologic: lymphopenia (38%), neutropenia (23%), anemia (20%) and leukopenia (13%); Nonhematologic: hyperglycemia, increased alanine aminotransferase, hypophosphatemia and hypertension (6% each)	Not reported	3.1 (n = 2)	44 (at least 1 drug); 28 (carfilzomib)
**Car-BiRD**								
Car-BiRD [47]	2	Transplant-eligible and -ineligible NDMM	72	Most common toxicities were low-grade gastrointestinal events; incidence of hematologic toxicities was noted to be very low across protocol	Most common event was infection (17%)	Not reported	Not reported	Not reported
**KCRd**								
UK NCRI Myeloma XI [48]	3	Transplant-eligible NDMM	526 (KCRd arm)	Not reported	Neutropenia: 16%; Anemia: 10%; Thrombocytopenia: 8%	Not reported	4.8	Dose modification to any agent: 64.6
**KRd plus daratumumab**								
MMY1001 [49]	1b	Transplant-eligible and -ineligible NDMM	22	IRRs (all grade 1 or 2) in 27% of patients	Most common (>10%) events included lymphopenia (64%), neutropenia (18%), diarrhea (18%), and pulmonary embolism (14%)	46	Not reported	Not reported
NCT03290950 (KRd+DARA) [51]	2	NDMM	20 (completed ≥1 cycle)	Not reported	Grade >3 AEs were hypotension, musculoskeletal deformity, back pain, dyspnea, lung infection, and febrile neutropenia	Not reported	Not reported	5 patients had dose reductions of LEN

*AE* adverse event, *ASCT* autologous stem cell transplantation, *Car-BiRD* carfilzomib induction with lenalidomide and clarithromycin consolidation and lenalidomide maintenance, *CFZ* carfilzomib, *CYKLONE* cyclophosphamide, carfilzomib, thalidomide and dexamethasone, *D* day, *DEX* dexamethasone, *IFM* Intergroupe francophone du myélome, *IRR* infusion-related reactions, *KCRd* carfilzomib, cyclophosphamide, lenalidomide, and dexamethasone, *KRd* carfilzomib, lenalidomide, and dexamethasone, *KTd* carfilzomib, thalidomide, and dexamethasone, *LEN* lenalidomide, *MMRC* Multiple Myeloma Research Consortium, *NDMM* newly diagnosed multiple myeloma, *NIH* National Institutes of Health, *PD* progressive disease, *SAE* serious AE, *UK NCR*I United Kingdom National Cancer Research Institute

**Table 4:** Adverse events of interest for carfilzomib–immunomodulatory drug combination therapies in patients with newly diagnosed multiple myeloma

Study (reference)	Phase	Patient population	Safety population, *n*	Hematologic events	Peripheral neuropathy events	Cardiovascular events
**KRd**						
MMRC (KRd with or without ASCT) [27]	1/2	Transplant-eligible and -ineligible NDMM	53	Any-grade during induction: Thrombocytopenia (68%), anemia (60%), and neutropenia (30%) Grade 3/4 during induction: Thrombocytopenia:(17%), anemia (21%), and neutropenia (17%)	Any-grade during induction: 23% (no grade ≥3 events, majority attributable to lenalidomide); No treatment discontinuations due to PN	Any-grade dyspnea during induction: 15%; Grade 3/4 dyspnea during induction: 4% Dyspnea was noted to occur early in treatment and was associated with fluid overload. Adjustment of hydration resulted in lower rates of dyspnea and no grade ≥3 events were observed in phase 2.
MMRC subset analysis [28]	1/2	Transplant-eligible and -ineligible NDMM, age ≥65 years	23	Grade 3/4 during induction: Thrombocytopenia:(39%),lymphopenia (35%), neutropenia (30%), and anemia (26%);	Any-grade attributable to KRd during induction: 22% (no grade 3 or 4 events)	Not reported
MMRC (KRd+ASCT) [33]	2	Transplant-eligible NDMM	76	Any-grade related to KRd: thrombocytopenia (57%), lymphopenia (39%), anemia (39%), neutropenia (28%); Grade 3/4: lymphopenia (28%), neutropenia (18%)	Any-grade related to KRd: 39%	Pretransplant, 2 of 71 patients had asymptomatic decrease in ejection fraction
KRd+LEN maintenance [30]	2	Transplant-eligible and -ineligible NDMM	45	Any-grade: lymphopenia (100%); thrombocytopenia (93%); leukopenia (82%); anemia (73%), neutropenia (67%)	Any-grade: 38% (no grade 3 or 4 events, including at 5-year follow up); No treatment discontinuations due to PN	Any-grade cardiac events: 38%; Grade 3 cardiac events (no grade 4 events reported) were: congestive heart failure (*n* = 2) and hypertension (*n* = 3)
IFM study; KRd+ASCT+LEN maintenance [35]	2	Transplant-eligible NDMM, age <65 years	46	Induction (grade 3/4): neutropenia (13%), lymphopenia (11%), thrombocytopenia (2%), anemia (2%); Consolidation (grade 3/4): neutropenia (29%), lymphopenia (41.5%), thrombocytopenia (17%)	No grade 3/4 PN reported	Serious cardiac and vascular disorder AEs (17%); There were 25 cardiac and vascular events of any grade
MRD response-driven KRd [36]	1/2	NDMM	29	Grade 3/4: lymphopenia (41%), leukopenia (7%), neutropenia (3%), and thrombocytopenia (3%)	Not reported	Grade 3/4 cardiopulmonary events: 7%; 1 patient discontinued due to myocardial infarction
FORTE; KRd versus KCd [38]	3	Transplant-eligible NDMM, age <65 years	KRd+ASCT: 158; KRd 12 cycles: 157	At least 1 grade 3/4 or serious AE: KRd+ASCT: 22%; KRd 12 cycles: 18%	Not reported	Grade 3/4 or serious event: cardiac (KRd+ASCT, 3%; KRd 12 cycles, 2%), hypertension (KRd+ASCT, 3%; KRd 12 cycles, 8%), DVT/PE (KRd+ASCT, 1%; KRd 12 cycles, 3%)
**KTd**						
Carthadex [41]	2	Transplant-eligible NDMM, age 18–65 years	91	Blood and lymphatic system disorders (over all cycles): 16% (any grade), 7% (grade 3 or 4), 3% (serious event)	Polyneuropathy (over all cycles): 40% (any grade), 1% (grade 3 or 4), 2% (serious event); 47 of 59 polyneuropathy events reported were considered at least possibly related to thalidomide; 1 patient with a carfilzomib dose reduction due to polyneuropathy	Cardiac disorders (over all cycles): 19% (any grade), 5% (grade 3; no grade 4 events reported), 5% (serious event); Vascular disorders (over all cycles): 30% (any grade), 7% (grade 3/4), 2% (serious event); Grade 3 cardiac disorder events reported included heart failure (*n* = 3), dyspnea (*n* = 1), and chest pain (*n* = 1)
**CYKLONE**						
CYKLONE [44]	1b/2	Transplant-eligible NDMM	64	Any-grade: neutropenia (55%), anemia (44%), lymphopenia (42%), leukopenia (39%); Grade ≥3: lymphopenia (38%), neutropenia (23%), anemia (20%), leukopenia (13%)	Any-grade: 31%; Grade ≥3: 0; All events were considered predominantly related to thalidomide; No treatment discontinuations due to PN	Any-grade: cardiac events (16%), dyspnea (20%), hypertension (9%); Grade ≥3: cardiac events (6%), dyspnea (3%), hypertension (6%); Grade ≥3 cardiac events included heart failure, chest pain, atrial fibrillation, conduction disorder, restrictive cardiomyopathy, and ventricular tachycardia
**Car-BiRD**						
Car-BiRD [47]	2	Transplant-eligible and -ineligible NDMM	72	The rate of treatment-emergent hematologic toxicities was noted to be low	Any-grade: 31%; Grade ≥3: 0	Not reported
**KCRd**						
UK NCRI Myeloma XI [48]	3	Transplant-eligible NDMM	526 (KCRd arm)	Neutropenia: 11.5% (grade 3), 4.9% (grade 4); Anemia: 10.0% (grade 3), 0.2% (grade 4); Thrombocytopenia: 5.3% (grade 3), 3.1% (grade 4)	Grade 2–4 motor neuropathy: 2.7%^a^; Grade 2–4 sensory neuropathy: 2.5%^a^	Arrhythmias: 2.3%^a^; Heart failure: 1%^a^; Hypertension: <1%^a^
**KRd plus daratumumab**						
MMY1001 [49]	1b	Transplant-eligible and -ineligible NDMM	22	Grade 3/4 events: lymphopenia (64%), neutropenia (18%)	Not reported	One patient reported transient grade 3 cardiac failure. The patient resumed treatment at CFZ 56 mg/m^2^; There was no change from baseline in median left ventricular ejection fraction
NCT03290950 (KRd+DARA) [51]	2	NDMM	28	Grade >3 AEs included febrile neutropenia	Not reported	Not reported

*AE* adverse event, *ASCT* autologous stem cell transplantation, *Car-BiRD* carfilzomib induction with lenalidomide and clarithromycin consolidation and lenalidomide maintenance, *CFZ* carfilzomib, *CYKLONE* cyclophosphamide, carfilzomib, thalidomide, and dexamethasone, *D* day, *DEX* dexamethasone, *IFM* Intergroupe francophone du myélome, *KCRd* carfilzomib, cyclophosphamide, lenalidomide, and dexamethasone; *KRd* carfilzomib, lenalidomide, and dexamethasone, *KTd* carfilzomib, thalidomide, and dexamethasone, *LEN* lenalidomide, *MMRC* Multiple Myeloma Research Consortium, *NDMM* newly diagnosed multiple myeloma, *NIH* National Institutes of Health, *PD* progressive disease, *UK NCRI* United Kingdom National Cancer Research Institute

^a^Rates estimated from bar graphs

#### Carfilzomib–thalidomide–dexamethasone

CARTHADEX was a phase 1/2 trial that evaluated carfilzomib–thalidomide–dexamethasone (KTd) as induction and consolidation therapy in transplant-eligible NDMM patients. During KTd induction, patients received four cycles of carfilzomib 20/27, 20/36, 20/45, or 20/56 mg/m^2^. In the overall population (*N* = 91), KTd induction resulted in an ORR of 90%, including a CR in 25% of patients and a ≥VGPR (primary endpoint) in 68% of patients [41]. Response rates improved with transplant and KTd consolidation (Table 2). After consolidation, patients with standard- and high-risk disease showed no difference in ≥VGPR rates and had similar CR rates. At a median follow-up of 23 months, the median PFS was not reached and the 3-year PFS rate was 72%. A subsequent update of the CARTHADEX trial assessed the impact of prolonged KTd induction therapy (eight cycles) at carfilzomib 56 mg/m^2^ on depth of response [42]. Patients treated with prolonged KTd induction (*n* = 26) had higher ≥VGPR and ≥CR rates at end of induction compared with those treated with four KTd induction cycles at 56 mg/m^2^ (*n* = 20). However, depth of response between the two groups was noted to be similar after completion of ASCT and consolidation [42]. Another update of the CARTHADEX trial found that postconsolidation sCR and VGPR rates were numerically higher for the 36–56 versus 27 mg/m^2^ carfilzomib dose levels, but that PFS/OS were similar between these doses [43].

#### Carfilzomib–cyclophosphamide–thalidomide–dexamethasone

The phase 1/2 CYKLONE trial (*N* = 64) examined a four-drug regimen termed CYKLONE (carfilzomib–cyclophosphamide–thalidomide–dexamethasone) for the treatment of transplant-eligible NDMM patients [44]. The MTD of carfilzomib in CYKLONE was 20/36 mg/m^2^ (phase 1 primary endpoint). The ORR across all treatment cycles and dose levels was 91%. At the MTD, the ≥VGPR rate after four cycles was 59% (phase 2 primary endpoint).

The safety of carfilzomib–thalidomide combinations (KTd and CYKLONE) was consistent with previously reported safety of both agents. Lack of overlapping toxicities (especially neuropathy) between these two agents allowed a favorable safety profile for these regimens.

#### Carfilzomib–clarithromycin–lenalidomide–dexamethasone

Combination treatment with the antibiotic clarithromycin plus a PI has been found preclinically to induce synergistic cytotoxicity [45, 46]. The phase 2 Car-BiRD study (*N* = 72) of carfilzomib–clarithromycin–lenalidomide–dexamethasone (Car-BiRD) evaluated carfilzomib as part of a sequential treatment strategy: Kd induction (until maximal response) followed by BiRD consolidation and lenalidomide maintenance for transplant-eligible or -ineligible NDMM patients [47]. Carfilzomib (30-min IV infusion) was given for the first 3 weeks of each 4-week cycle at a dose of 20/45 mg/m^2^ (first 26 patients) or 20/56 mg/m^2^ (thereafter). Kd induction resulted in an ORR of 91%, ≥VGPR rate of 71%, and a ≥CR rate of 13%. Following BiRD consolidation, ORR, ≥VGPR, and ≥CR rates improved to 100%, 95%, and 28%, respectively. Lenalidomide maintenance further improved depth of response in 19% of patients.

#### Carfilzomib–cyclophosphamide–lenalidomide–dexamethasone

In the phase 3 UK NCRI Myeloma XI trial, transplant-eligible patients were randomized to the upfront PI-based quadruplet induction regimen of carfilzomib–cyclophosphamide–lenalidomide–dexamethasone (KCRd), or triplet induction therapy (cyclophosphamide–lenalidomide–dexamethasone [CRd] or cyclophosphamide–thalidomide–dexamethasone [CTd]) [48]. Patients in the triplet induction arms could have received a second induction with bortezomib–cyclophosphamide–dexamethasone (VCd) before ASCT depending on post first induction maximum response (≥VGPR, no second induction; PR or minor response, randomized to VCd or nothing; stable or progressive disease, VCd as second induction). Carfilzomib 20/36 mg/m^2^ was administered twice weekly for the first 3 weeks of each 4-week cycle. A total of 1056 patients underwent induction randomization (CTd, *n* = 265; CRd, *n* = 265; KCRd, *n* = 526). Deeper responses with carfilzomib-based quadruplet versus IMiD-based triplet treatment were observed at the end of first induction: the ≥VGPR rate at end of initial induction was 82.3% for KCRd versus 52.8 and 64.9% for CTd and CRd, respectively. This difference in depth of response was sustained until ≥100 days post-ASCT.

The quadruplet combination was well tolerated in this large phase 3 trial [48]. Grade ≥3 neutropenia occurred in 16% of KCRd patients, compared with 13% for CTd and 22% for CRd. Grade ≥2 sensory neuropathy rates were lower in the KCRd and CRd arms compared with the CTd arm (~2% vs ~10%). The incidences of any-grade cardiac failure and hypertension in the KCRd arm were both <2%.

#### Carfilzomib–lenalidomide–dexamethasone plus daratumumab

The phase 1b MMY1001 study evaluated KRd-daratumumab in NDMM patients [49, 50]. Patients were treated with this combination for ≤ 13 treatment cycles or until discontinuation for ASCT. Carfilzomib (20/70 mg/m^2^) was administered once weekly. This combination (*n* = 21) resulted in an ORR of 100% with 91 and 57% of patients achieving ≥VGPR and ≥CR, respectively. The 1-year PFS rate was 95%.

An ongoing phase 2 trial is evaluating MRD using blood and bone marrow-based assays after KRd-daratumumab treatment for NDMM. Preliminary results showed the feasibility of longitudinal MRD tracking in three patients who completed the MRD assays [51].

The phase 2 ASCENT trial (NCT03289299) is assessing the feasibility of KRd-daratumumab induction for high-risk SMM.

### Safety of carfilzomib–IMiD combinations in NDMM

The safety profile of carfilzomib has been well established in RRMM, where it has shown to have manageable AEs [53] and a favorable benefit-risk profile given the observed PFS/OS advantage. In this setting, the most common grade 3/4 AEs associated with single-agent carfilzomib were primarily hematologic [54]. Cardiovascular events have also been associated with carfilzomib, with hypertension (mainly grades 1 and 2) being the most common type. In a pooled analysis of phases 1–3 RRMM clinical studies (*n* = 2044 carfilzomib-exposed patients), any-grade and grade ≥3 hypertension rates were 18.5% and 5.9%, respectively, and any-grade and grade ≥3 cardiac failure rates were 6.7% and 4.4%, respectively [55]. Carfilzomib-associated cardiovascular events have been found to be generally reversible and manageable, as reflected by relatively low carfilzomib discontinuation rates across studies [55–57]. Furthermore, the efficacy benefit of carfilzomib in terms of reduction in the risk of progression/death has been found to outweigh the risks observed with carfilzomib (including cardiac failure or hypertension) among RRMM patients [55].

Carfilzomib has been associated with low rates of peripheral neuropathy (PN) in RRMM, an AE associated with bortezomib that may impair QoL and limit treatment duration [58]. The low rate of PN with carfilzomib is consistent with preclinical results showing that in contrast to bortezomib, carfilzomib has minimal activity against nonproteasomal proteases, including the stress-induced, prosurvival protease HtrA2/Omi [8]. This selectivity may explain the difference in PN incidence between the carfilzomib and bortezomib arms in ENDEAVOR [19]. In ASPIRE, addition of carfilzomib to Rd did not increase PN rates [24].

The safety profile of carfilzomib–IMiD combination therapy in NDMM patients has been consistent with the known safety profile of carfilzomib. Below, we summarize the above AEs of interest observed in the large, randomized NDMM studies (FORTE and Myeloma XI). General safety and AEs of interest for all NDMM studies are shown in Tables 3 and 4.

#### Hematologic adverse events

Hematologic events were the most common AEs observed in frontline clinical trials of carfilzomib–IMiD combinations (Table 4). The largest datasets come from the FORTE and Myeloma XI trials. In the FORTE trial, 18–22% of KRd patients had ≥1 grade ≥3 or serious hematologic AE [38]. Hematologic AE rates were comparable for KRd and KCd (18–22% vs 17%). In the KCRd arm of the Myeloma XI trial, rates of grade ≥3 neutropenia and thrombocytopenia were 16.4%, and 8.4%, respectively [48].

#### Peripheral neuropathy

Consistent with findings in RRMM, carfilzomib-based combination therapy has been associated with a low incidence of grade ≥2 PN in NDMM and a low rate of carfilzomib discontinuation or dose reductions due to PN (Table 4). In the KCRd arm of the Myeloma XI trial, grade ≥2 motor and sensory neuropathy rates were ~3% each [48]. PN rates have not been reported for the FORTE trial.

#### Cardiovascular events

Cardiovascular events have been reported with carfilzomib–IMiD combinations in the frontline setting (Table 4). In the FORTE trial, the rate of grade 3/4 or serious cardiac events was 2–3% in the KRd arms and 3% in the KCd arm [38]. Hypertension (grade 3/4, or serious in severity) was reported in 8% of patients who received 12 KRd cycles and in 3% patients who received KRd or KCd with ASCT [38]. In the KCRd versus CRd versus CTd arms of the phase 3 Myeloma XI study, any-grade arrhythmia, heart failure, and hypertension were reported in approximately 2.3% versus 1% versus 1.5%, 1% versus 0% versus 0%, and <1% versus <1% versus <1% of patients [48].

Although cardiovascular events have been observed with carfilzomib, they appear to be manageable and reversible in the majority of cases [56]. Importantly, grade ≥3 heart failure rates are relatively low considering carfilzomib efficacy. Clinical experience with carfilzomib has aided development of strategies for managing and mitigating cardiovascular events [56, 57]; clinical guidelines have been developed in collaboration with cardiologists to minimize cardiotoxicity with carfilzomib treatment [59].

## Conclusions

Carfilzomib-based, PI–IMiD combination therapies have been shown to be highly efficacious with a favorable safety profile in NDMM, as evidenced by the data from several studies (single-arm and randomized) reviewed above. These regimens elicit deep and durable responses, including MRD negativity, which have translated to high PFS and OS rates. Furthermore, a consistent benefit for these regimens has been observed across patient segments, including in both transplant-eligible and -ineligible patients, across age subgroups, and regardless of cytogenetic risk. To date, results have been reported from only one randomized phase 3 study of carfilzomib-based IMID combination therapy in NDMM, and there are currently no phase 3 data for how these therapies directly compare with the most recent frontline standards of care. Future data from such studies will be important for evaluating the role of carfilzomib-based, PI–IMiD combination therapy in NDMM.

Bortezomib-based, PI–IMiD triplet combinations are considered standards of care in the frontline setting. For example, VRd is considered a preferred regimen by the NCCN for transplant-eligible and -ineligible patients based on data showing that this regimen improved PFS and OS compared with Rd. Despite the absence of controlled and randomized trials, carfilzomib-based, PI–IMiD combinations may offer several advantages relative to this standard of care. For some patients, PN associated with bortezomib may interrupt treatment, resulting in suboptimal response and QoL. These patients may benefit from a carfilzomib-based PI–IMiD regimen. Although there are currently no head-to-head data of carfilzomib- versus bortezomib-based, PI–IMiD therapy in NDMM, KRd (without or without ASCT) is one of the most active frontline regimens observed in NDMM studies [27–36, 38–40, 60]. Preliminary data from the observational CoMMpass study (NCT01454297) suggest that improved response rates and longer event-free survival are associated with KRd versus VRd in NDMM [61], while other preliminary data from CoMMpass and the Flatiron observational study suggest longer treatment duration with VRd [62]. However, these findings need to be validated in a prospective manner. The ongoing, randomized phase 3 ENDURANCE study (NCT01863550; *N* = 1000+), which is evaluating KRd versus VRd, will provide important head-to-head data regarding the benefit-risk profiles of carfilzomib- versus bortezomib-based PI–IMiD therapy in NDMM, with the limitation of capping carfilzomib treatment duration to nine cycles.

Carfilzomib has been associated with manageable and reversible cardiovascular events in RRMM, and these events merit monitoring in NDMM. In a meta-analysis of NDMM and RRMM patients (*N* = 2594), any-grade and grade ≥3 congestive heart failure rates were 4.1% and 2.5%, respectively [63].

The introduction of new antimyeloma agents has significantly improved the outlook of MM patients; however, MM remains an incurable disease. To achieve long-term remissions or even functional cure for NDMM patients, the most potent agents need to be used upfront. Based on favorable clinical outcomes of carfilzomib therapies in the relapsed setting, we believe that a carfilzomib–IMiD combination will also benefit NDMM patients. Ongoing frontline studies are evaluating the impact of adding new drugs with novel mechanisms, such as monoclonal antibodies, to the carfilzomib–IMiD combination to achieve functional cures in MM.
